# Quantitative Assessment of Late Gadolinium Enhancement and Edema at Cardiac Magnetic Resonance in Low-Risk Myocarditis Patients

**DOI:** 10.3390/tomography8020078

**Published:** 2022-04-01

**Authors:** Caterina Beatrice Monti, Francesco Secchi, Marco Alì, Francesco Saverio Carbone, Luca Bonomo, Davide Capra, Nazanin Mobini, Giovanni Di Leo, Francesco Sardanelli

**Affiliations:** 1 Department of Biomedical Sciences for Health, Università degli Studi di Milano, 20133 Milan, Italy; caterina.monti@unimi.it (C.B.M.); davide.capra@unimi.it (D.C.); nazanin.mobini@unimi.it (N.M.); francesco.sardanelli@unimi.it (F.S.); 2 Unit of Radiology, IRCCS Policlinico San Donato, 20097 San Donato Milanese, Italy; gianni.dileo77@gmail.com; 3 Department of Diagnostic Imaging and Stereotactic Radiosurgery, C.D.I. Centro Diagnostico Italiano S.p.A., 20147 Milan, Italy; marco.ali@cdi.it; 4 Bracco Imaging S.p.A., 20134 Milan, Italy; 5 Corso di Laurea in Medicina e Chirurgia, Università degli Studi di Milano, Via Festa del Perdono 7, 20122 Milan, Italy; carbone.francescosaverio@gmail.com (F.S.C.); luca.bonomo@eoc.ch (L.B.)

**Keywords:** myocarditis, edema, magnetic resonance imaging, gadolinium

## Abstract

In this study, we aimed to quantify LGE and edema at short-tau inversion recovery sequences on cardiac magnetic resonance (CMR) in patients with myocarditis. We retrospectively evaluated CMR examinations performed during the acute phase and at follow-up. Forty-seven patients were eligible for retrospective LGE assessment, and, among them, twenty-five patients were eligible for edema evaluation. Both groups were paired with age- and sex-matched controls. The median left ventricle LGE was 6.4% (interquartile range 5.0–9.2%) at the acute phase, 4.4% (3.3–7.2%) at follow-up, and 4.3% (3.0–5.3%) in controls, the acute phase being higher than both follow-up and controls (*p <* 0.001 for both), while follow-up and controls did not differ (*p =* 0.139). An optimal threshold of 5.0% was obtained for LGE with 87% sensitivity and 48% specificity; the positive likelihood ratio (LR) was 1.67, and the negative LR was 0.27. Edema was 12.8% (9.4–18.1%) at the acute phase, 7.3% (5.5–8.8%) at follow-up, and 6.7% (5.6–8.6%) in controls, the acute phase being higher than both follow-up and controls (both *p <* 0.001), while follow-up and controls did not differ (*p =* 0.900). An optimal threshold of 9.5% was obtained for edema with a sensitivity of 76% and a specificity of 88%; the positive LR was 6.33, and the negative LR was 0.27. LGE and edema thresholds are useful in cases of suspected mild myocarditis.

## 1. Introduction

Myocarditis is an inflammatory disease of the myocardium, defined by histological and immunohistochemical criteria [1,2,3]. Its onset can be acute, subacute, or chronic, and it may either be focal or involve the myocardium diffusely [4]. The most common cause of myocarditis is a viral infection, followed by bacterial infection, drugs cardiotoxicity, and autoimmune disorders [5,6,7]. The incidence of myocarditis is unknown due to the heterogeneity of its clinical features and the widespread differences in its diagnostic workup [8,9]. One study published by Vos et al. [10] in 2015, based on the International Classification of Diseases, estimated myocarditis’ global prevalence to be approximately 22/10,000 people per year between 1990 and 2013, but underestimation is highly likely [11].

The reference standard for the diagnosis of myocarditis is an endomyocardial biopsy. However, it is rarely performed due to its risks, which makes it inadvisable in patients with mild, low-risk disease [1,3,12]. In addition, the sensitivity of endomyocardial biopsy depends on the area where myocarditis occurs and whether it is correctly identified. Several non-invasive diagnostic examinations are available for the workup of patients with suspected myocarditis. Among them, cardiac magnetic resonance (CMR) enables the evaluation of cardiac morphology and function and the characterization of myocardial tissue [1]. Indeed, myocarditis is also one of the most accepted indications for CMR [13], particularly in patients with suspected mild disease, namely those without hemodynamic compromise or heart failure, whereas patients with severe disease usually undergo endomyocardial biopsy [14].

CMR diagnosis of myocarditis is currently based on the Lake Louise criteria [15], which state that two out of three CMR findings (edema, early gadolinium enhancement, and late gadolinium enhancement (LGE)) must be present to finalize such diagnosis. Three CMR sequences allow for ruling in or ruling out the diagnosis of myocarditis according to the following criteria: T2-weighted inversion-recovery magnitude, T1-weighted inversion recovery, and bright-blood steady-state free precession sequences. The first is useful for detecting edema throughout the myocardium, the second for assessing LGE, and the third may show biventricular kinetic alterations. The criteria combination with the best performance is represented by edema together with LGE, with 78% accuracy, 67% sensitivity, and 91% specificity [16].

Lake Louise criteria have, so far, been assessed only visually, meaning edema and LGE are not quantified in clinical practice. Indeed, while qualitative differences can be appreciated between myocarditis patients and healthy individuals, no quantitative variation has been determined, and no diagnostic thresholds for quantitative scoring are currently available.

Thus, the aim of our study was to analyze and quantify edema and LGE in myocarditis patients both at the acute phase and follow-up as well as in controls, in order to establish quantitative thresholds for such parameters, under the hypothesis of significantly higher values of those biomarkers in myocarditis patients. This would allow for a more accurate diagnostic interpretation, particularly for uncertain cases.

## 2. Materials and Methods

### 2.1. Study Design and Population

The local Ethics Committee approved this retrospective study (Ethics Committee of San Raffaele Clinical Research Hospital; protocol code “CardioRetro”, number 122/int/2017; approved on 14 September 2017 and amended on 2 February 2021). This study was partially supported by Ricerca Corrente funding from the Italian Ministry of Health to IRCCS Policlinico San Donato. This research received no specific grant from any public funding agency or the commercial or non-profit sectors. Due to the retrospective nature of this study, specific, informed consent was waived. All CMR examinations included in this study were performed at our institution between September 2008 and March 2017. Patients were selected for clinical suspicion of myocarditis, represented by the presence of cardiac signs or symptoms (such as hypotension or syncope), biomarkers (troponin elevation), changes in electrocardiographic pattern (e.g., sinus tachycardia associated with nonspecific ST/T-wave changes, pathological Q wave, or wide QRS [17]), arrhythmia, or left ventricular dysfunction, with the exclusion of other underlying cardiac diseases. Moreover, such patients had a CMR examination that was compatible with acute myocarditis instead of another cardiac disease and a follow-up CMR examination consistent with a history of myocarditis. Such findings were deemed consistent with a diagnosis of acute myocarditis at the first CMR examination. Patients with non-mild disease, represented by a subnormal ejection fraction (<55%), were excluded.

Inclusion criteria for LGE evaluation in myocarditis patients were the availability of one CMR examination at the acute phase and one at follow-up, both comprising sequences for LGE evaluation. We also included CMRs of age- and sex-matched controls, who were to have a CMR examination for non-malignant, occasional arrhythmias that were deemed negative by an expert (over 10 years of CMR experience) radiologist’s report.

Inclusion criteria for edema in myocarditis patients were the presence of one CMR examination at the acute phase and one at follow-up, both comprising turbo inversion recovery magnitude sequences. We also included CMRs of age- and sex-matched controls, who were to have a CMR examination that was deemed negative by an expert radiologist’s report.

### 2.2. Image Acquisition

Before September 2014, CMR was performed with a 1.5-T unit (Magnetom Sonata, Siemens Medical Solutions, Erlangen, Germany) with 40-mT/m gradient power and a 4-channel surface phased-array coil; after September 2014, CMR was performed with another 1.5-T unit (Magnetom Aera, Siemens Medical Solutions, Erlangen, Germany), with 45-mT/m gradient power and an 18-channel surface phased-array coil. Coils were placed over the thorax of patients in the supine position. Prospective gating was always applied to the electrocardiographic signal.

On both machines, for LGE evaluation, short-axis sequences were acquired 5–15 min after injecting 0.1–0.15 mmol/kg of gadobutrol (Gadovist, Bayer Healthcare, Leverkusen, Germany). While the echo time (TE) was 3.33 ms, the repetition time (TR) was adapted to the heart rate, the inversion time was chosen from 260 to 330 ms to better differentiate the myocardium, and the flip angle was 25°. For the evaluation of myocardial edema, T2-weighted short-axis short-tau inversion recovery (STIR) sequences, with patient-dependent TR and TE, an inversion time of 150 ms, and a flip angle of 180° were used. Cine sequences were bright-blood steady-state free precession sequences, with a TE of 1.40 ms, TR of 48.30 ms, and a flip angle of 60°. STIR sequences were always run before administering contrast, while cine sequences were run after contrast administration. An 8-mm slice thickness was always chosen.

### 2.3. Image Analysis

**Figure 1:**
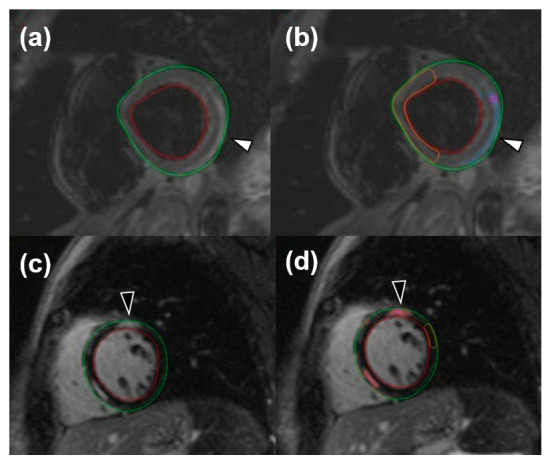
Short-tau inversion recovery images for the visualization of edema (white arrowheads) both without (**a**) and with (**b**) an automatic quantification mask of remote myocardium and late gadolinium enhancement (black arrowheads), without (**c**) and with (**d**) an automatic quantification mask of remote myocardium.

For image analysis, the QMass post-processing tool (Medis Suite MR Software, Medis Medical Imaging Systems, Leiden, The Netherlands) was used. Cine sequences were manually bordered to obtain left ventricle functional data such as end-diastolic volume index, end-systolic volume index, stroke volume, and ejection fraction. Short-axis turbo inversion recovery magnitude sequences were also manually bordered; a threshold of 2 standard deviations (SD) was used to identify edema. Short-axis LGE sequences were manually bordered, and a 6-SD threshold was used to identify enhanced areas. Image analysis for edema was performed twice by one reader and once by a different reader. An example of automatic LGE and edema quantification can be viewed in Figure 1.

### 2.4. Diagnostic Criterion

After data collection and analysis, a diagnostic algorithm for myocarditis was hypothesized for patients with LGE and edema data. The LGE and edema of patients were assessed, and when the two parameters were concordant (either both above the threshold found through receiver operating characteristics [ROC] analysis or both below), the diagnosis of myocarditis was either finalized or excluded. When the LGE and edema were discordant, a visual assessment was performed to discern whether the two phenomena were anatomically related. If there was at least one area where edema and LGE were concomitantly present, the criterion was deemed positive; otherwise, it was regarded as negative because an aberrant quantification of one of the two parameters could have been due to faults in automatic tissue characterization. The criterion was tested by each reader with their edema data.

### 2.5. Statistical Analysis

Data were reported as median with their interquartile range (IQR). Differences were investigated for significance with non-parametric tests, either with the Wilcoxon test when paired or the Mann-Whitney U test when independent. ROC analysis was performed for edema and LGE data comparing the acute phase to controls to set thresholds and evaluate their sensitivity and specificity. A diagnosis of myocarditis, based on a clinical suspicion of myocarditis represented by the presence of cardiac signs or symptoms or rise of cardiac biomarkers in the absence of other cardiac pathologies, and a CMR examination, positive according to the Lake Louise criteria, was used as ground truth for ROC analysis. Thresholds maximizing sensitivity and specificity (minimum total number of false positive and false negative cases) were chosen. Positive and negative likelihood ratios were calculated. The reproducibility of the diagnostic criterion was assessed through Bland-Altman analysis and reported as bias and its coefficient of repeatability (CoR). Concordance of the diagnostic criteria was assessed through Cohen’s κ.

Statistical analysis was performed using SPSS v.17.0 (IBM SPSS Inc., Chicago, IL, USA). Whenever a statistical test yielded a *p*-value < 0.05, the null hypothesis was rejected.

## 3. Results

### 3.1. Study Population

**Figure 2:**
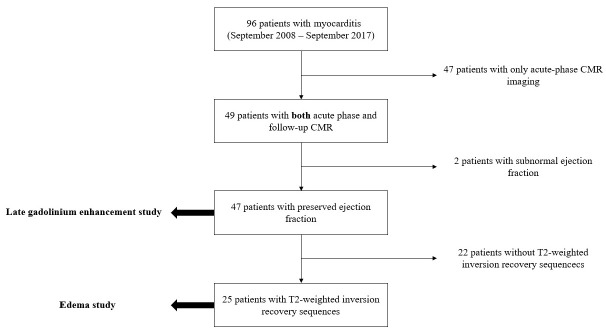
Flowchart describing the process of patient selection throughout our study. CMR, cardiac magnetic resonance.

From a total of 96 patients with suspected myocarditis and a CMR examination performed between September 2008 and March 2017 retrieved from our database, 47 were eligible for the LGE study, of which 25 were eligible for the edema study according to the inclusion and exclusion criteria, as shown in Figure 2.

### 3.2. LGE Group

Results for LGE are shown in Table 1. Both myocarditis patients and controls were comprised of 36 males and 11 females. The age of myocarditis patients (median 31 years, IQR 20–43) and of controls (median 31 years, IQR 20–43) were comparable (*p =* 0.868). Four patient acquisitions were performed on Magnetom Sonata, 41 were performed on Magnetom Aera, and controls were paired accordingly.

No significant differences were found in the end-diastolic volume index, end-systolic volume index, stroke volume, and ejection fraction among acute phase patients, follow-up patients, and controls.

The left ventricle LGE percentages were 6.4% (IQR 5.0–9.2%) at the acute phase, 4.4% (IQR 3.3–7.2%) at follow-up, and 4.3% (IQR 3.0–5.3%) in controls. Significant differences could be found between the acute phase and follow-up (*p <* 0.001) as well as between the acute phase and controls (*p <* 0.001), while the difference between follow-up and controls was not significant (*p =* 0.139).

**Figure 3:**
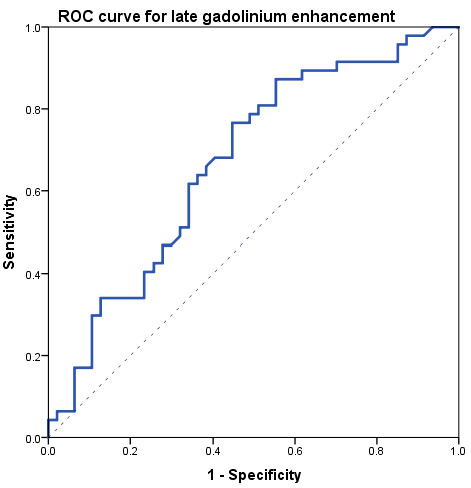
Receiver operating characteristics (ROC) curve obtained comparing late gadolinium enhancement data between acute-phase and controls.

At ROC analysis (Figure 3), a ROC curve was obtained for LGE analysis between the acute phase and controls, resulting in an area under the curve of 0.68 and a threshold of 5.0% LGE, with a sensitivity of 87% (41/47) and a specificity of 48% (22/47). The positive likelihood ratio for LGE was 1.67, while the negative likelihood ratio was 0.27.

### 3.3. Edema Group

Edema data is shown in Table 2. Both myocarditis patients and controls comprised 18 males and 7 females. The age of myocarditis patients (median 35 years, IQR 26–45) and of controls (median 35 years, IQR 24–44) were comparable (*p =* 0.954).

No significant differences were found within the end-diastolic volume index, end-systolic volume index, stroke volume, and ejection fraction among acute phase patients, follow-up patients, and controls.

Left ventricle edema percentages were 12.8% (IQR 9.4–18.1%) at the acute phase, 7.3% (IQR 5.5–8.8%) at follow-up, and 6.7% (IQR 5.6–8.6%) in controls. Significant differences were found between the acute phase and follow-up or controls (*p <* 0.001 for both), while the difference between follow-up and controls was not significant (*p =* 0.900).

**Figure 4:**
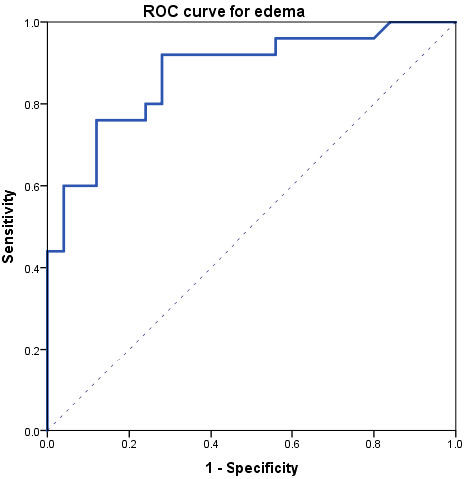
Receiver operating characteristics (ROC) curve obtained comparing edema data between acute-phase and controls.

At ROC analysis (Figure 4), edema data comparing the acute phase and controls resulted in an area under the curve of 0.88, with an optimal threshold of 9.5% edema, a sensitivity of 76% (19/25), and a specificity of 88% (22/25). The positive likelihood ratio for edema was 6.33, while the negative likelihood ratio was 0.27.

The intra-reader reproducibility of edema measurement was 84% with a bias of 0.45% and a CoR of 1.86%, while the inter-reader reproducibility was 75% with a bias of 0.54% and a CoR of 2.86%.

### 3.4. Diagnostic Criteria

The application of the proposed diagnostic criteria showed 24 true positives, 22 true negatives, 4 false positives, and 4 false negatives, leading to a sensitivity of 92% and a specificity of 85%, with a positive likelihood ratio of 6.00 and a negative likelihood ratio of 0.09. Intra-reader Cohen’s κ was 1.000 (*p <* 0.001), while inter-reader Cohen’s κ was 0.766 (*p <* 0.001).

## 4. Discussion

In this study, we tried to establish a quantitative threshold for both LGE and edema in low-risk myocarditis patients. Both analyzed study populations matched known epidemiologic data for patients commonly affected by myocarditis. In fact, Kytö et al. [18] reported that myocarditis was more common in men (77%) than in women (23%), and the median age of myocarditis patients was 33 years (IQR 23–50).

During the acute phase of myocarditis, LGE represents areas with acute inflammation, while later, it indicates residual scarring [19,20]. Our LGE analysis showed that LGE passed from a median value of 6.4% to a median value of 4.4%, a substantial reduction of about 30%. Interestingly, the percentual myocardial volume with a signal attributable to LGE in the follow-up with our patients was not significantly different from that of the controls: even though some residual enhancement, probably due to fibrosis, was present at follow-up, mild myocarditis survivors bear a relatively low amount of residual scarring. The presence of about 4% of LGE in controls can be explained by signal inhomogeneities or artifacts, which are detected by our semi-automatic software even when visual assessment deems the CMR examination negative for LGE.

**Figure 5:**
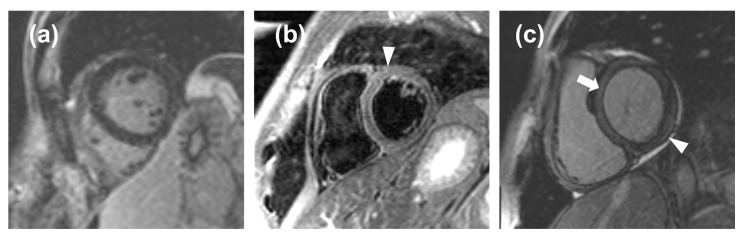
(**a**), a false negative case in which the noise masked the late gadolinium enhancement (LGE); (**b**), a false positive case in which the noise mimicked edema (arrowhead); (**c**), a false positive case in which an inaccurate inversion time caused a relative hyperintensity of the myocardium (arrow) that mimicked a thin, subepicardial band of LGE (arrowhead).

The ROC curve obtained from LGE data from acute-phase patients and controls has a moderate area under the curve, resulting in an optimal threshold of 5% with a high value of sensitivity (87%) but low specificity (48%). Indeed, minimal LGE appears most commonly as a thin, subepicardial line, easily mistaken for adipose tissue, that the software cannot assess with sufficient accuracy, resulting in a falsely negative assessment (Figure 5). This means that quantitative LGE evaluation may be useful for excluding myocarditis in patients testing negative for our threshold, with a negative likelihood ratio of 0.27, which indicates that a negative LGE test determines a reduction of the post-test probability of the disease by 73% as compared to the pre-test probability [21]. Hence, LGE could be used as a quantitative criterion, albeit with certain limitations with regards to its specificity and visual assessment. Our LGE values are lower than those obtained by Ferreira et al. [19] for both acute-phase patients (11%, IQR 5% to 21%) and controls (0%, IQR 0% to 2%), as well as those obtained by Luetkens et al. [22] at the acute phase (15.8 ± 12.0%) and follow-up (7.2 ± 5.9%), whereas our controls LGE values are similar to those reported by Luetkens et al. [22] for controls (4.8 ± 4.4%). Since LGE values for the acute phase and follow-up depend on disease severity, we should highlight that other studies did not exclude patients with a compromised ejection fraction, hence displaying higher values compared to ours, which were derived from patients with mild myocarditis with preserved ejection fraction.

Regarding edema analysis, there were no significant differences between the left ventricular functional parameters of patients and controls, which is consistent with the age-matching we performed. The left ventricular ejection fraction of patients was well within the normality range, meaning that patients had mild disease without severe impairment of cardiac function. This was expected since such patients were at low risk, with no hemodynamic compromise or heart failure.

The significant differences in edema observed between the acute phase and follow-up or controls may prove that edema, representing acute inflammation, is higher during the onset and initial stages of myocarditis, while it tends to reduce at follow-up. The ROC curve obtained from edema data of acute-phase patients and controls has a good area under the curve, resulting in an acceptable value of sensitivity (76%) and a high value of specificity (88%). The positive likelihood ratio for (6.33) edema suggests that it could be used to finalize a diagnosis of myocarditis, with a high increase in disease probability when edema tests positive. The negative likelihood ratio for edema (0.27) suggests that it would yield an adequate performance when excluding myocarditis, equal to that of LGE. Therefore, myocardial edema could be used as a quantitative biomarker to be added to visual assessment. Moreover, for quantification of edema, it is notable that the percentage of myocardial volume attributable to edema in controls (median 6.7%) was not significantly different from that of low-risk myocarditis at follow-up (median 7.3%). This finding can be explained by the fact that inflammation is expected to recede in the time intercurrent between the acute phase and follow-up and null at follow-up. The presence of some degree of edema detected in the controls is likely due to signal inhomogeneities or artifacts (Figure 5), which are detected by our semi-automatic software, causing a falsely positive assessment even when the visual assessment by an expert radiologist deems the CMR examination negative for edema. Indeed, as reported by h-Ici et al. [23], the sensitivity and specificity of T2-weighted acquisition for edema assessments are 95% and 78%, respectively. The wish for the highest automatization of LGE and edema quantification for the inclusion of such processes in routine clinical workflows led to the search for new thresholds that could be utilized to deem fully automated LGE or edema quantification positive or negative for myocarditis. In this regard, raising the SD thresholds would not have proven beneficial, as it would have led to a loss of detection of areas where LGE or edema was indeed present.

The proposed quantitative diagnostic criterion for myocarditis provides both high sensitivity (92%) and high specificity (85%), with good positive and negative likelihood ratios (6.00 and 0.09 respectively), therefore, bearing a higher sensitivity than a visual assessment of edema and LGE as a part of Lake Louise Criteria (sensitivity 25%, specificity 95%), with only a slightly lower specificity [24]. Therefore, such quantitative criteria could be used for diagnosing or excluding myocarditis in patients undergoing CMR as a part of their clinical workflow, leading to a smaller number of false positives. Future developments may add the assessment of mapping sequences and extracellular volume to further improve sensitivity and specificity [25].

Our study had some limitations, the first being its retrospective nature. Moreover, it was monocentric, meaning the results are in relation to the magnetic resonance units used on our population and to the chosen sequences, technical parameters, and the type and dosage of contrast material. In addition, our subjects were studied using two different 1.5-T magnetic resonance units and coils. Further studies should be conducted to confirm our results, especially in terms of diagnostic thresholds.

## 5. Conclusions

In conclusion, the results of our study show that quantitative thresholds for LGE and edema can be defined for helping in ruling in and ruling out myocarditis to be primarily used when visual assessment is inconclusive.

## Figures and Tables

**Table 1:** Results of late gadolinium enhancement evaluation.

	Acute	Follow-Up	Controls	p_acute/follow-up_	p_acute/controls_	p_follow-up/controls_
Age (years)	31 (20–43)	31 (20–43)	31 (20–43)	-	0.868	0.868
Males (%)	77	77	77	-	-	-
EDVI (mL/m^2^)	74 (61–84)	72 (63–79)	73 (65–82)	0.898	0.742	0.525
ESVI (mL/m^2^)	25 (19–29)	23 (20–29)	24 (20–29)	0.331	0.719	0.702
SV (mL)	89 (75–100)	90 (77–101)	84 (76–104)	0.488	0.988	0.853
EF (%)	67 (61–69)	66 (63–70)	67 (60–70)	0.208	0.617	0.877
LGE (%)	6.44 (5.01–9.15)	4.39 (3.28–7.22)	4.29 (3.00–5.25)	<0.001 *	<0.001 *	0.139

Data are reported as median and interquartile ranges. EDVI, end-diastolic volume index; ESVI, end-systolic volume index; SV, stroke volume; EF, ejection fraction; LGE, late gadolinium enhancement. * indicates statistical significance.

**Table 2:** Results of edema evaluation.

	Acute	Follow-Up	Controls	p_acute/follow-up_	p_acute/controls_	p_follow-up/controls_
Age (years)	35 (26–45)	35 (26–45)	35 (24–44)	-	0.954	0.954
Males (%)	28	28	28	-	-	-
EDVI (mL/m^2^)	76 (61–85)	73 (63–79)	75 (68–84)	0.927	0.676	0.331
ESVI (mL/m^2^)	25 (19–28)	25 (22–29)	24 (21–29)	0.692	0.884	0.690
SV (mL)	91 (76–104)	96 (80–103)	91 (79–113)	0.943	0.634	0.793
EF (%)	67 (62–69)	65 (63–68)	68 (64–71)	0.637	0.465	0.150
Edema (%)	12.76 (9.36–18.07)	7.29 (5.48–8.82)	6.67 (5.57–8.63)	<0.001 *	<0.001 *	0.900

Data are reported as median and interquartile ranges. EDVI, end-diastolic volume index; ESVI, end-systolic volume index; SV, stroke volume; EF, ejection fraction. * indicates statistical significance.

## Data Availability

Raw data is available upon reasonable request to the corresponding author.
